# Prevalence and sociodemographic correlates of marriage among adolescent mothers in Canada, 1989–2018

**DOI:** 10.17269/s41997-022-00728-3

**Published:** 2022-12-23

**Authors:** Andrée-Anne Fafard St-Germain, Karen Busby, Marcelo L. Urquia

**Affiliations:** 1 Manitoba Centre for Health Policy, Community Health Sciences, Max Rady College of Medicine, Rady Faculty of Health Sciences, University of Manitoba, Winnipeg, Manitoba Canada; 2 Department of Nutritional Sciences, University of Toronto, Toronto, Ontario Canada; 3 Faculty of Law, University of Manitoba, Winnipeg, Manitoba Canada; 4 Dalla Lana School of Public Health, University of Toronto, Toronto, Ontario Canada

**Keywords:** Child marriage, Teen birth, Parental age gap, Foreign-born status, Neighbourhood income, Mariage d’enfants, naissances chez les adolescentes, différence d’âge des parents, naissance à l’étranger, revenu selon le quartier

## Abstract

**Objectives:**

Female marriage before age 18 is a global health issue related to gender inequality, but it is understudied in Canada. This study examined marriage trends among mothers aged < 18 versus older mothers and the sociodemographic correlates of marriage among adolescent mothers aged < 18 and older adolescent mothers.

**Methods:**

Using the Canadian Vital Statistics – Birth Database, marriage prevalence was estimated by maternal age groups (< 18-year, 18–19-year, 20–24-year, and 25–49-year) between 1989–1990 and 2017–2018 (*n* = 10,399,250). Multivariable logistic regression was then used to examine the sociodemographic characteristics associated with marriage within adolescent maternal age group (< 18-year, 18–19-year, and 20–24-year) among births registered between 2000 and 2018.

**Results:**

From 1989–1990 to 2017–2018, marriage prevalence declined 80.5%, 60.2%, 47.3%, and 16.0% in the < 18-year, 18–19-year, 20–24-year, and 25–49-year groups, respectively. Within the < 18-year, 18–19-year, and 20–24-year adolescent maternal age groups, older maternal age, larger parental age gap, foreign-born parents, rurality, and earlier birth period were associated with higher adjusted odds of marriage. Higher maternal neighbourhood income was associated with marriage among births to mothers aged 18–19 and 20–24 years but not among those to mothers aged < 18 years.

**Conclusion:**

Marriage prevalence declined among mothers of all ages, but the shifts away from marriage appear stronger among younger mothers. The sociodemographic correlates of marriage are generally similar among mothers below age 18 and slightly older adolescent mothers.

## Résumé

**Objectifs:**

Le mariage des filles de moins de 18 ans est un problème de santé mondial lié aux inégalités entre les sexes, mais il est insuffisamment étudié au Canada. Notre étude porte sur les tendances du mariage chez les mères de < 18 ans comparativement aux mères plus âgées et sur les corrélats sociodémographiques du mariage chez les mères adolescentes de < 18 ans et les mères adolescentes plus âgées.

**Méthode:**

À l’aide de la Base canadienne de données de l’état civil – Naissance, nous avons estimé la prévalence des mariages selon le groupe d’âge maternel (< 18 ans, 18-19 ans, 20-24 ans et 25-49 ans) entre 1989-1990 et 2017-2018 (n = 10 399 250). Au moyen d’une analyse de régression logistique multivariée, nous avons ensuite examiné les caractéristiques sociodémographiques associées au mariage dans les groupes d’âge des mères adolescentes (< 18 ans, 18-19 ans et 20-24 ans) pour les naissances enregistrées entre 2000 et 2018.

**Résultats:**

De 1989-1990 à 2017-2018, la prévalence des mariages a baissé de 80,5 %, 60,2 %, 47,3 % et 16,0 % dans les groupes de < 18 ans, de 18-19 ans, de 20-24 ans et de 25-49 ans, respectivement. Dans les groupes d’âge des mères adolescentes de < 18 ans, de 18-19 ans et de 20-24 ans, un âge maternel plus avancé, une plus grande différence d’âge des parents, la naissance des parents à l’étranger, la ruralité et la période de naissance plus précoce étaient associées à une probabilité de mariage ajustée plus élevée. Le revenu maternel plus élevé selon le quartier était associé au mariage pour les naissances de mères de 18-19 et de 20-24 ans, mais pas chez les mères de < 18 ans.

**Conclusion:**

La prévalence du mariage a baissé chez les mères de tout âge, mais l’abandon du mariage semble plus prononcé chez les mères plus jeunes. Les corrélats sociodémographiques du mariage sont généralement semblables chez les mères de moins de 18 ans et les mères adolescentes légèrement plus âgées.

## Introduction

Marriage has repeatedly been shown to be associated with positive health outcomes for women and their children (Brown, 2010; Kane, 2016; Kiernan & Pickett, 2006; Luo et al., 2004; Shah et al., 2011; Shapiro et al., 2018). Research indicates the health advantages associated with marriage are likely driven by a higher propensity to adopt health-promoting attitudes and behaviours, and by selection into marriage (Brown, 2010; Kane, 2016; Kiernan & Pickett, 2006). However, global evidence suggests that girl child marriage, defined as a union or marriage of a female below age 18 (Efevbera & Bhabha, 2020), is adversely associated with maternal and child health (Efevbera et al., 2017; Marphatia et al., 2017; Raj et al., 2010). These adverse associations seem to be partly or fully due to sociodemographic and economic differences between those who marry before age 18 and those who marry at an older age (Efevbera et al., 2017; Raj et al., 2010). International conventions consider child marriage a human rights violation (General Assembly of the United Nations, 2018; UNICEF, 2020). Through the adoption of the 2030 Agenda for Sustainable Development, all United Nations Member States, including Canada, committed to help end child marriage to promote gender equality (United Nations, 2020). Child marriage is currently legal in Canada, but in the absence of systematic monitoring, little is known about the sociodemographic distribution of female marriage below age 18 domestically (Koski & Clark, 2021).

The age of majority of 18 or 19 years is the legal age to marry in every province and territory, but marriage below the legal age is permitted because of statutory exemptions, such as parental consent and court approval (Canadian Legal Information Institute, 2021). Prior to June 2015, there was no explicit federal legislation regarding the minimum age below which marriage was fully proscribed (Government of Canada, 2014a, b). Other than in Quebec and Ontario where marriage below age 16 was completely prohibited, all other provinces and territories had a minimum age requirement of 15 or 16 years but permitted marriage below these age thresholds if specific exemptions were met, such as having court approval or proof of a pregnancy and parental consent (Canadian Legal Information Institute, 2021). In June 2015, the federal government established a national minimum age requirement of 16 years for marriage and created a new criminal offence for those who knowingly contribute to or celebrate a marriage involving an individual below that age (Canadian Legal Information Institute, 2021; Government of Canada, 2022). Under this new federal legislation, individuals who are 16 and 17 years are still permitted to marry in every province and territory with parental consent and/or court approval.

Based on census data, it is estimated that less than 1% of Canadians aged 15 to 17 years were married or in common-law unions between 2001 and 2016 (Koski & Clark, 2021). Child marriage prevalence was found to be higher among girls than among boys and to differ by age, nativity and Indigenous status, and region of residence (Koski & Clark, 2021). An examination of marriage certificates issued in five provinces also signals a decline in the incidence of child marriage since 2000 (Koski & Clark, 2021). To date, it is unclear whether the sociodemographic selection into marriage differs between female adolescents below age 18 and those of older ages. Better understanding the sociodemographic context of marriage among Canadian females below age 18 is important to guide future domestic actions towards child marriage and gender equality as per the Agenda for Sustainable Development.

This study utilized birth registration data to first describe national trends in marriage prevalence among mothers below age 18 and among older mothers from 1989–1990 to 2017–2018. Then, focusing on birth registrations from 2000 to 2018, the sociodemographic patterning of marriage among births to mothers below age 18 was characterized and contrasted with the patterns observed among births to slightly older adolescent mothers aged 18–19 and 20–24 years (Sawyer et al., 2018).

## Methods

### Data source and study sample

This study used data from the 1989 to 2018 Canadian Vital Statistics – Birth Database (CVSB). This administrative database includes all annual live births registered in provincial and territorial vital statistics registries, except for births that occurred in the Yukon in 2017 and 2018 (Health Statistics Division, 2019). It contains information on parental sociodemographic characteristics, including age, nativity status, and marital status.

**Fig. 1:**
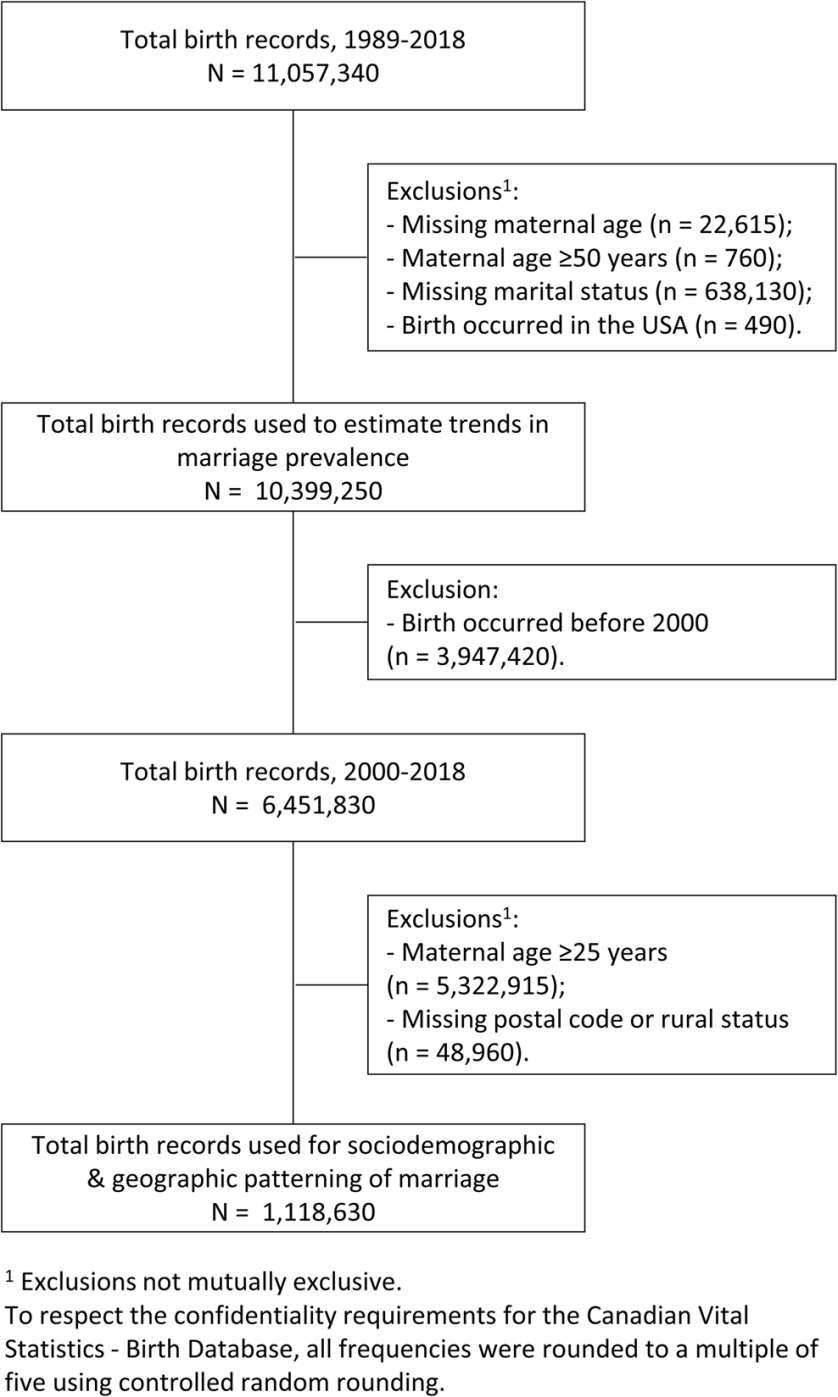
Sample selection process

To describe trends in marriage prevalence, the study sample included all births to mothers aged ≤ 49 years that occurred in Canada from January 1, 1989 to December 31, 2018 and had complete information on maternal age and marital status (*n* = 10,399,250). To characterize and contrast the sociodemographic patterning of marriage, the sample was limited to births with adolescent mothers aged ≤ 24 (Sawyer et al., 2018); that were registered since 2000 because of better consistency in geographic information availability using the Postal Code Conversion File Plus (PCCF+) (Health Statistics Division, 2019); and with complete data on maternal age, marital status, postal code, and rural status (*n* = 1,118,630). Figure 1 presents the sample selection process.

### Marital status and maternal age

The binary indicator for marital status was primarily determined using maternal conjugal status at the time of the delivery of the registered birth. Mothers classified as single, widowed, divorced, or separated were coded as unmarried, while mothers classified as married were coded as such. Widowed, divorced, or separated mothers were grouped with those unmarried, because most reported being unmarried to the father when information on parental marital relationship was available. For observations with missing information for maternal conjugal status but with available parental marital relationship, the latter was used to categorize mothers as married or unmarried. Since mothers in common-law unions are processed in the CVSB as having unknown conjugal status in provinces collecting this information (Health Statistics Division, 2019), these were either categorized using parental marital relationship or excluded from the sample due to missing marital status.

Maternal age was expressed in completed years based on the mother’s most recent birthday prior to the delivery of the registered birth. It was categorized as < 18, 18–19, 20–24, and 25–49 years. As births to mothers aged 25–49 years represent nearly 80% of the total sample, they provided a reference trend for marriage prevalence in Canada. To examine the sociodemographic correlates of marriage, births to adolescent mothers aged 18–19 and 20–24 years were used as comparison groups to interpret results among births to adolescent mothers aged < 18 years, which is consistent with the conceptualization of adolescence (Sawyer et al., 2018) and research on adolescent pregnancies (Chen et al., 2007; Ganchimeg et al., 2014).

### Sociodemographic characteristics

The selection of sociodemographic characteristics was informed by the information available in the CVSB and by international (Efevbera et al., 2017; Kamal et al., 2015; Marphatia et al., 2017; Raj et al., 2010, 2014) and domestic (Koski & Clark, 2021) research on child marriage. The variables included maternal and paternal nativity status (Canadian-born or foreign-born); parental age difference equal to paternal age minus maternal age (fathers < 2, 2–4, or ≥ 5 years older than mothers); region of residence (Atlantic, Quebec, Ontario, Prairies, British Columbia, or territories); rural status identifying mothers residing in communities with fewer than 10,000 inhabitants (rural and small town or urban) (Statistics Canada, 2009, 2018); neighbourhood income quintiles derived for each Census Metropolitan Area, Census Area, or provincial residual areas using information on before-tax household income within Dissemination Areas (Statistics Canada, 2009, 2018); and birth period (2000–2004, 2005–2009, 2010–2014, or 2015–2018). Maternal age was also included to differentiate younger and older mothers within each adolescent age group (≤ 15 or 16–17 years for the < 18-year group; 18 or 19 for the 18–19-year group; and 20, 21, 22, 23, or 24 for the 20–24-year group). To mitigate sample loss, an “unavailable” category was created for each variable when applicable. Due to collinearity between the unavailable category for parental age gap and unavailable category for paternal nativity status, the results for these two categories should be interpreted with caution in the adjusted analyses.

### Statistical analyses

Trends in marriage prevalence were estimated separately for birth to mothers aged < 18, 18–19, 20–24, and 25–49 years. Due to smaller cell sizes in the youngest age group, prevalence estimates were calculated over a 2-year period from 1989 to 2018. Frequencies and proportions for the sociodemographic characteristics were presented by marital status and adolescent maternal age group (< 18-year, 18–19-year, and 20–24-year) for births that occurred between 2000 and 2018. Logistic regression was used to estimate odds ratios (ORs) of marriage by sociodemographic characteristics within each adolescent maternal age group. Since several sociodemographic characteristics have the potential to covary with one another, multivariable logistic regression models were conducted to estimate adjusted odds ratios (AORs) of marriage.

To respect confidentiality requirements, all frequencies were rounded to a multiple of 5 using a controlled random rounding technique. All analyses were performed using SAS 9.4 (SAS Institute, Cary, NC).

## Results

**Fig. 2:**
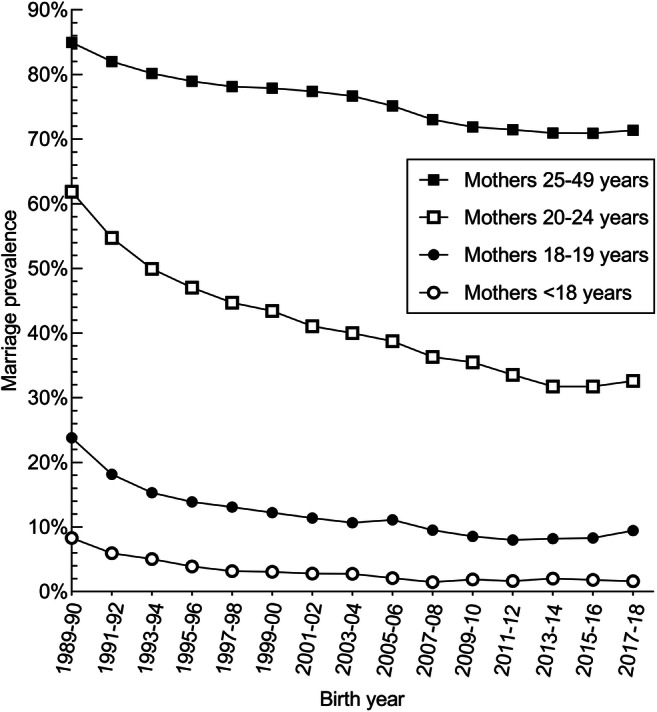
Marriage prevalence by maternal age group, 1989–1990 to 2017–2018

Between 1989 and 2018, marriage prevalence estimates were highest among births to mothers aged 25–49 years, followed by births to mothers aged 20–24, 18–19, and < 18 years (Fig. 2). Overall, marriage prevalence declined over time in all maternal age groups, but a steeper decline occurred prior to 2001–2002 in the 20–24-year, 18–19-year, and < 18-year groups. From 1989–1990 to 2017–2018, marriage prevalence dropped by 13.6, 29.3, 14.3, and 6.7 percentage points in the 25–49-year, 20–24-year, 18–19-year, and < 18-year maternal age groups, respectively; these drops represent a 16.0%, 47.3%, 60.2%, and 80.5% decline relative to the 1989–1990 estimate.

Among births recorded between 2000 and 2018 with available postal code and rural status, the proportion of births to married mothers was 36.3%, 9.7%, and 2.1% in the 20–24-year, 18–19-year, and < 18-year maternal age groups, respectively (Table 1). Within all three adolescent maternal age groups, a higher proportion of marital births had older mothers, foreign-born mothers or fathers, larger parental age gap, and mothers residing in Ontario or in urban areas. The proportion of marital births also declined between the 2000–2004 and 2015–2018 periods.

**Table 1:** Sociodemographic characteristics by marital status and adolescent maternal age group, 2000–2018

Sociodemographic characteristics	Maternal age group < 18-year		Maternal age group 18–19-year		Maternal age group 20–24-year	
	Unmarried	Married	Unmarried	Married	Unmarried	Married
	*N* = 64,535	*N* = 1410	*N* = 139,620	*N* = 15,080	*N* = 571,630	*N* = 326,355
	*n* (%)	*n* (%)	*n* (%)	*n* (%)	*n* (%)	*n* (%)
Maternal age in years						
≤ 15	8790 (13.6)	40 (2.8)	---	---	---	---
16–17	55,745 (86.4)	1370 (97.2)	---	---	---	---
18	---	---	57,580 (41.2)	4080 (27.1)	---	---
19	---	---	82,040 (58.8)	11,000 (72.9)	---	---
20	---	---	---	---	98,800 (17.3)	22,485 (6.9)
21	---	---	---	---	108,860 (19.0)	38,000 (11.6)
22	---	---	---	---	116,140 (20.3)	59,215 (18.1)
23	---	---	---	---	120,985 (21.2)	86,035 (26.4)
24	---	---	---	---	126,845 (22.2)	120,620 (37.0)
Parental age gap						
< 2 years	13,850 (21.5)	145 (10.3)	34,705 (24.9)	2580 (17.1)	159,390 (27.9)	85,010 (26.0)
2–4 years	19,435 (30.1)	485 (34.4)	38,820 (27.8)	5130 (34.0)	154,375 (27.0)	106,810 (32.7)
≥ 5 years	10,410 (16.1)	690 (48.9)	31,770 (22.8)	6940 (46.0)	164,100 (28.7)	130,855 (40.1)
Unavailable	20,840 (32.3)	90 (6.4)	34,325 (24.6)	430 (2.9)	93,765 (16.4)	3680 (1.1)
Maternal nativity status						
Canadian-born	61,335 (95.0)	785 (55.7)	131,665 (94.3)	8060 (53.4)	532,090 (93.1)	196,855 (60.3)
Foreign-born	2820 (4.4)	580 (41.1)	7040 (5.0)	6670 (44.2)	35,080 (6.1)	123,845 (37.9)
Unavailable	380 (0.6)	45 (3.2)	920 (0.7)	345 (2.3)	4460 (0.8)	5655 (1.7)
Paternal nativity status						
Canadian-born	40,565 (62.9)	665 (47.2)	96,770 (69.3)	7150 (47.4)	433,800 (75.9)	186,455 (57.1)
Foreign-born	2755 (4.3)	625 (44.3)	7465 (5.3)	7130 (47.3)	38,400 (6.7)	126,800 (38.9)
Unavailable	21,215 (32.9)	120 (8.5)	35,390 (25.3)	795 (5.3)	99,430 (17.4)	13,100 (4.0)
Maternal region of residence						
Atlantic	6475 (10.0)	60 (4.3)	15,100 (10.8)	610 (4.0)	62,460 (10.9)	16,990 (5.2)
Quebec	9010 (14.0)	185 (13.1)	28,975 (20.8)	2280 (15.1)	178,520 (31.2)	45,740 (14.0)
Ontario	15,940 (24.7)	565 (40.1)	30,295 (21.7)	5610 (37.2)	104,975 (18.4)	122,955 (37.7)
Prairies	24,840 (38.5)	425 (30.1)	48,450 (34.7)	4890 (32.4)	158,125 (27.7)	98,550 (30.2)
British Columbia	6475 (10.0)	165 (11.7)	14,140 (10.1)	1645 (10.9)	59,910 (10.5)	41,255 (12.6)
Territories	1795 (2.8)	10 (0.7)	2660 (1.9)	45 (0.3)	7640 (1.3)	865 (0.3)
Rurality of maternal residence						
Urban	40,040 (62.0)	1035 (73.4)	93,090 (66.7)	11,315 (75.0)	400,510 (70.1)	250,165 (76.7)
Rural or small town	24,495 (38.0)	375 (26.6)	46,530 (33.3)	3765 (25.0)	171,120 (29.9)	76,190 (23.3)
Neighbourhood income quintiles						
Quintile 1 (lowest)	27,160 (42.1)	595 (42.2)	54,995 (39.4)	5635 (37.4)	196,760 (34.4)	95,130 (29.1)
Quintile 2	12,985 (20.1)	315 (22.3)	29,550 (21.2)	3615 (24.0)	126,280 (22.1)	77,825 (23.8)
Quintile 3	9680 (15.0)	210 (14.9)	22,265 (15.9)	2445 (16.2)	101,780 (17.8)	64,050 (19.6)
Quintile 4	7805 (12.1)	165 (11.7)	17,950 (12.9)	1955 (13.0)	82,705 (14.5)	51,380 (15.7)
Quintile 5 (highest)	5760 (8.9)	115 (8.2)	12,885 (9.2)	1310 (8.7)	56,670 (9.9)	34,795 (10.7)
Unavailable	1145 (1.8)	10 (0.7)	1975 (1.4)	120 (0.8)	7435 (1.3)	3175 (1.0)
Birth period						
Birth year 2000–2004	20,235 (31.4)	600 (42.6)	41,970 (30.1)	5350 (35.5)	148,970 (26.1)	103,590 (31.7)
Birth year 2005–2009	20,295 (31.4)	370 (26.2)	41,555 (29.8)	4615 (30.6)	161,815 (28.3)	95,640 (29.3)
Birth year 2010–2014	16,350 (25.3)	305 (21.6)	36,870 (26.4)	3255 (21.6)	156,530 (27.4)	77,735 (23.8)
Birth year 2015–2018	7655 (11.9)	135 (9.6)	19,230 (13.8)	1855 (12.3)	104,315 (18.2)	49,390 (15.1)

--- not applicable

Due to vetting procedures at Statistics Canada’s Research Data Centres, all frequencies were rounded to a multiple of 5 using a controlled random rounding technique. Column percent may not add to 100% due to rounding

Within each adolescent maternal age group, the AORs of marriage declined substantially with younger maternal age and increased with larger parental age gaps, with the greatest increase in the < 18-year group (Table 2). AORs of marriage were substantially higher among births to foreign-born compared to Canadian-born mothers in all three maternal age groups. Unavailable maternal nativity status was also associated with elevated AORs of marriage within each maternal age group. AORs of marriage were elevated among births with foreign-born fathers within each maternal age group, and the association tended to be stronger in the < 18-year and 18–19-year groups.

**Table 2:** Unadjusted and adjusted odds ratios of marriage by sociodemographic characteristics within adolescent maternal age group, 2000–2018

Sociodemographic characteristics	Maternal age group < 18-year		Maternal age group 18–19-year		Maternal age group 20–24-year	
	OR (95%CI)	AOR^a^ (95%CI)	OR (95%CI)	AOR^a^ (95%CI)	OR (95%CI)	AOR^a^ (95%CI)
Maternal age in years						
≤ 15	0.19 (0.14–0.26)	0.25 (0.18–0.34)	---	---	---	---
16–17	1.00	1.00	---	---	---	---
18	---	---	0.53 (0.51–0.55)	0.54 (0.51–0.56)	---	---
19	---	---	1.00	1.00	---	---
20	---	---	---	---	0.24 (0.24–0.24)	0.22 (0.21–0.22)
21	---	---	---	---	0.37 (0.36–0.38)	0.34 (0.33–0.34)
22	---	---	---	---	0.54 (0.53–0.55)	0.51 (0.50–0.52)
23	---	---	---	---	0.75 (0.74–0.76)	0.73 (0.72–0.74)
24	---	---	---	---	1.00	1.00
Parental age gap						
< 2 years	1.00	1.00	1.00	1.00	1.00	1.00
2–4 years	2.38 (1.97–2.87)	2.22 (1.83–2.69)	1.78 (1.69–1.87)	1.69 (1.60–1.79)	1.30 (1.29–1.31)	1.32 (1.30–1.33)
≥ 5 years	6.33 (5.28–7.58)	4.33 (3.58–5.23)	2.94 (2.80–3.08)	2.04 (1.94–2.16)	1.50 (1.48–1.52)	1.08 (1.07–1.10)
Unavailable	0.41 (0.31–0.53)	0.39 (0.25–0.61)	0.17 (0.15–0.19)	0.12 (0.11–0.15)	0.07 (0.07–0.07)	0.04 (0.03–0.04)
Maternal nativity status						
Canadian-born	1.00	1.00	1.00	1.00	1.00	1.00
Foreign-born	16.07 (14.34–18.01)	6.17 (5.27–7.23)	15.48 (14.87–16.09)	6.36 (6.02–6.72)	9.54 (9.42–9.66)	5.18 (5.09–5.28)
Unavailable	9.25 (6.74–12.70)	9.70 (6.60–14.27)	6.13 (5.41–6.95)	7.83 (6.71–9.14)	3.43 (3.30–3.57)	5.90 (5.61–6.20)
Paternal nativity status						
Canadian-born	1.00	1.00	1.00	1.00	1.00	1.00
Foreign-born	13.84 (12.33–15.54)	3.97 (3.37–4.67)	12.93 (12.42–13.46)	4.40 (4.17–4.64)	7.68 (7.58–7.78)	2.98 (2.93–3.04)
Unavailable	0.35 (0.29–0.43)	1.28 (0.87–1.88)	0.30 (0.28–0.32)	1.62 (1.41–1.85)	0.31 (0.30–0.32)	1.96 (1.88–2.04)
Maternal region of residence						
Atlantic	0.54 (0.41–0.71)	0.65 (0.49–0.85)	0.40 (0.37–0.44)	0.49 (0.45–0.54)	0.44 (0.43–0.45)	0.49 (0.48–0.50)
Quebec	1.20 (1.01–1.43)	0.70 (0.57–0.85)	0.78 (0.74–0.82)	0.51 (0.48–0.55)	0.41 (0.40–0.42)	0.27 (0.27–0.28)
Ontario	2.07 (1.82–2.35)	1.41 (1.22–1.64)	1.83 (1.76–1.91)	1.23 (1.17–1.30)	1.88 (1.86–1.90)	1.35 (1.33–1.37)
Prairies	1.00	1.00	1.00	1.00	1.00	1.00
British Columbia	1.49 (1.24–1.79)	1.19 (0.98–1.45)	1.15 (1.08–1.22)	0.98 (0.92–1.05)	1.10 (1.08–1.12)	0.87 (0.86–0.89)
Territories	0.33 (0.18–0.62)	0.49 (0.25–0.95)	0.17 (0.13–0.23)	0.29 (0.21–0.39)	0.18 (0.17–0.19)	0.26 (0.24–0.28)
Rurality of maternal residence						
Urban	1.00	1.00	1.00	1.00	1.00	1.00
Rural or small town	0.59 (0.52–0.66)	1.31 (1.14–1.51)	0.67 (0.64–0.70)	1.32 (1.26–1.39)	0.71 (0.70–0.72)	1.27 (1.25–1.28)
Maternal neighbourhood income quintile						
Quintile 1	1.00	1.00	1.00	1.00	1.00	1.00
Quintile 2	1.11 (0.97–1.27)	1.15 (0.99–1.34)	1.19 (1.14–1.24)	1.31 (1.24–1.38)	1.27 (1.26–1.29)	1.45 (1.43–1.47)
Quintile 3	0.99 (0.84–1.16)	1.07 (0.90–1.27)	1.07 (1.02–1.12)	1.21 (1.14–1.28)	1.30 (1.28–1.32)	1.61 (1.58–1.63)
Quintile 4	0.96 (0.81–1.14)	1.14 (0.95–1.38)	1.06 (1.00–1.12)	1.34 (1.26–1.43)	1.28 (1.26–1.30)	1.68 (1.65–1.71)
Quintile 5	0.91 (0.74–1.11)	1.14 (0.91–1.41)	0.99 (0.93–1.05)	1.34 (1.24–1.44)	1.27 (1.25–1.29)	1.72 (1.68–1.75)
Unavailable	0.40 (0.21–0.75)	0.56 (0.29–1.06)	0.59 (0.49–0.71)	0.76 (0.62–0.93)	0.88 (0.84–0.92)	0.98 (0.93–1.03)
Birth period						
Birth year 2000–2004	1.00	1.00	1.00	1.00	1.00	1.00
Birth year 2005–2009	0.61 (0.54–0.70)	0.65 (0.57–0.75)	0.87 (0.83–0.91)	0.81 (0.77–0.85)	0.81 (0.77–0.85)	0.73 (0.72–0.74)
Birth year 2010–2014	0.63 (0.55–0.72)	0.76 (0.66–0.89)	0.69 (0.66–0.72)	0.68 (0.64–0.72)	0.68 (0.64–0.72)	0.57 (0.56–0.58)
Birth year 2015–2018	0.59 (0.49–0.71)	0.67 (0.55–0.82)	0.76 (0.72–0.80)	0.69 (0.65–0.74)	0.69 (0.65–0.74)	0.52 (0.51–0.53)

--- not applicable; *AOR*, adjusted odds ratio; *OR*, odds ratio

^a^Adjusted for all sociodemographic characteristics listed in the table

Compared to births with mothers residing in the prairies, those with mothers residing in Ontario had higher AORs of marriage, while births with mothers residing in Quebec, the Atlantic provinces, and the territories had lower AORs in all three maternal age groups (Table 2). Births with mothers residing in British Columbia had lower AORs of marriage in the 20–24-year group, but the AORs were non-significant in the < 18-year and 18–19-year groups. In all three maternal age groups, maternal residence in a rural area or small town was associated with 27% to 32% higher adjusted odds of marriage compared to maternal residence in an urban area. No association was present between maternal neighbourhood income quintiles and marriage in the < 18-year maternal age group; the adjusted odds of marriage increased with higher neighbourhood income quintiles in the 18–19-year and 20–24-year groups. In all three maternal age groups, AORs of marriage were lower in more recent birth periods, and for births to mothers aged < 18 years, the largest decline occurred between 2000–2004 and 2005–2009.

## Discussion

Using national birth registrations from 1989 to 2018, this study documents a decrease in marriage prevalence among mothers aged < 18, 18–19, 20–24 and 25–49 years, and suggests a larger relative decline in prevalence in younger mothers, especially those below age 18. The study found that among adolescent mothers aged < 18, 18–19, and 20–24 years, older maternal age, larger parental age gap, maternal and paternal foreign-born status, rurality, and earlier birth period were associated with being married. Geographic variations were present in the adjusted odds of marriage, but the geographic patterning was similar across adolescent maternal age groups. While higher maternal neighbourhood income was associated with marriage among mothers aged 18–19 and 20–24 years, no association was present among mothers aged < 18 years.

Prior research relying on marriage certificates from five provinces found an overall decrease in marriage incidence between 2000 and 2018 among individuals below age 18 (Koski & Clark, 2021). Focusing on mothers below age 18, the current study shows a decline in marriage prevalence between 1989–1990 and 2017–2018, with the steepest decline occurring in the 1990s. It also highlights that marriage among mothers below age 18 has been uncommon for more than a decade in Canada, with a marriage prevalence near 2% or less since 2005–2006 and less than 5% since 1995–1996. By examining marriage prevalence in older mothers, the study suggests a general trend towards marriage becoming less common among mothers of all ages in Canada. However, the larger relative decline in marriage prevalence among younger mothers compared to those aged 25–49 years indicates a greater shift away from marriage in younger groups.

Research suggests that part of the health advantages associated with being married compared to being single or in a common-law union among adult women is due to selection into marriage, including selection by socioeconomic status (Brown, 2010; Kane, 2016). The current study indicates that sociodemographic selection into marriage is generally similar between mothers below age 18 and slightly older adolescent mothers, with the notable exception of maternal neighbourhood income. Higher socioeconomic status, as indicated by higher maternal neighbourhood income quintiles, was associated with marriage in older adolescent mothers but not mothers below age 18. This socioeconomic distinction between married mothers below age 18 and their older counterparts could contribute to difference in the health advantages of marriage over time. The current study also found that larger parental age gap was associated with marriage in all age groups, but the association was strongest among mothers below age 18. Greater age gaps between partners raise concerns for the well-being and development of young adolescent females as they may experience greater power imbalances and autonomy constraints (Marphatia et al., 2017). Finally, the similar result patterns by parental foreign-born status and maternal region of residence across adolescent maternal age groups suggest the potential role of sociocultural factors in influencing propensity to marry in Canada.

Some of the sociodemographic correlates of marriage among mothers below age 18 in this study are consistent with international research suggesting girl child marriage is more common among females living in rural areas, aged 16 or 17 compared to less than 16 years, and married to older husbands (Efevbera et al., 2017; Kamal et al., 2015; Marphatia et al., 2017; Raj et al., 2010, 2014). However, contrary to international research indicating an association between lower socioeconomic status and girl child marriage (Efevbera et al., 2017; Kamal et al., 2015; Marphatia et al., 2017; Raj et al., 2010, 2014), the current study found no association between neighbourhood income and marriage among mothers below age 18. This difference may be related to using a socioeconomic indicator measured at the area-level rather than the individual-level, but it may also suggest contextual differences in the factors associated with marriage among female adolescents below age 18.

### Limitations

This study focused on marriage among females having given birth in Canada, preventing generalization to all females. Given the interconnection between fertility and marriage, the marriage prevalence estimates among mothers below age 18 derived in this study are higher than those based on census data for females aged 15–17 years (Koski & Clark, 2021). The study likely underestimated marriage below age 18 among Canadian mothers, since some unmarried mothers below age 18 may have married after registering the birth but before turning 18. It was also not possible to identify mothers above the age of 18 who married before turning 18 because age at the time of marriage is not collected in the CVSB. Research suggests differences in maternal and infant health between mothers who are single versus those in a common-law union (Brown, 2010; Kiernan & Pickett, 2006; Luo et al., 2004; Shah et al., 2011; Shapiro et al., 2018), and there are indications that a growing proportion of unions involving Canadian youths aged 15–17 years are common-law unions rather than formal marriages (Koski & Clark, 2021). Due to unavailable data in the CVSB, it was impossible to differentiate unmarried single mothers from those in common-law unions. The sociodemographic characteristics included in this study were limited by the information available in the CVSB, and certain characteristics previously identified to be associated with early marriage in international studies, such as religion, education, and childhood socioeconomic circumstances (Kamal et al., 2015; Manning & Cohen, 2015; Marphatia et al., 2017; Raj et al., 2010), could not be examined.

## Conclusion

This study represents a novel examination of the prevalence and sociodemographic characteristics of marriage among mothers below the age of 18 and older adolescent mothers in Canada. It showed that marriage among mothers before age 18 has been uncommon for the last two decades and that the decline in marriage prevalence between 1989 and 2018 among mothers below age 18 was accompanied by a decline in marriage among older mothers. The results indicate that the sociodemographic correlates of marriage among adolescent mothers below age 18 are generally similar to those identified among slightly older adolescent mothers. Although female marriage before age 18 is generally uncommon in Canada, more research is needed to better understand its consequences and drivers domestically to continue promoting gender equality and supporting the agency of young female adolescents.

## Contributions to knowledge

What does this study add to existing knowledge?

- Marriage prevalence declined among mothers of all ages between 1989–1990 and 2017–2018, but the decline was steepest among mothers below age 18.
- The sociodemographic characteristics associated with marriage were generally similar among adolescent mothers below age 18 and those aged 18–19 and 20–24 years, except for higher neighbourhood income being associated with marriage only among older adolescent mothers.

What are the key implications for public health interventions, practice, or policy?

- Although marriage below age 18 is uncommon in Canada, it still occurs and must be examined separately from marriage at an older age. Considering Canada’s commitment to end child marriage to help achieve the Sustainable Development Goal of gender equality by 2030, additional research is needed to better understand the consequences and drivers of marriage before age 18 domestically to inform the development of effective actions.

## Data Availability

The data used in this study are available through the Canadian Research Data Centre’s Network upon receiving data access approval by Statistics Canada.

## Code availability

SAS statistical codes used for the analysis in this study can be shared upon request.

## References

[CR1] Brown SL (2010). Marriage and child well-being: Research and policy perspectives. Journal of Marriage and Family.

[CR2] Canadian Legal Information Institute. (2021). Canadian Legal Information Institute (CanLII). https://www.canlii.org/en/. Accessed January 30, 2021.

[CR3] Chen XK, Wen SW, Fleming N, Demissie K, Rhoads GG, Walker M (2007). Teenage pregnancy and adverse birth outcomes: A large population based retrospective cohort study. International Journal of Epidemiology.

[CR4] Efevbera Y, Bhabha J (2020). Defining and deconstructing girl child marriage and applications to global public health. BMC Public Health.

[CR5] Efevbera Y, Bhabha J, Farmer PE, Fink G (2017). Girl child marriage as a risk factor for early childhood development and stunting. Social Science & Medicine.

[CR6] Ganchimeg T, Ota E, Morisaki N, Laopaiboon M, Lumbiganon P, Zhang J (2014). Pregnancy and childbirth outcomes among adolescent mothers: A World Health Organization multicountry study. BJOG : An international Journal of Obstetrics and Gynaecology.

[CR7] General Assembly of the United Nations. (2018). A/C.3/73/L.22/Rev.1. Promotion and protection of the rights of children - Child, early and forced marriage. https://www.un.org/en/ga/third/73/proplist.shtml. Accessed March 8, 2022.

[CR8] Government of Canada. (2014a). Backgrounder: Comparative overview of proposed changes in the Zero Tolerance for Barbaric Cultural Practices Act. https://web.archive.org/web/20141106211137/http://news.gc.ca/web/article-en.do?mthd=tp&crtr.page=1&nid=900359&crtr.tp1D=930. Accessed March 8, 2022.

[CR9] Government of Canada. (2014b). Backgrounder: Zero Tolerance for Barbaric Cultural Practices Act: An overview. https://web.archive.org/web/20141106211144/http://news.gc.ca/web/article-en.do?mthd=tp&crtr.page=1&nid=900339&crtr.tp1D=930. Accessed March 8, 2022.

[CR10] Government of Canada. (2022). Civil Marriage Act SC 2005, c. 33. https://laws-lois.justice.gc.ca/eng/acts/c-31.5/20150618/P1TT3xt3.html. Accessed March 8, 2022.

[CR11] Health Statistics Division. (2019). *Canadian Vital Statistics Birth Database: Data dictionary and user guide, 2018*. Ottawa, ON: Health Statistics Division, Statistics Canada.

[CR12] Kamal SMM, Hassan CH, Alam GM, Ying Y (2015). Child marriage in Bangladesh: Trends and determinants. Journal of Biosocial Science.

[CR13] Kane JB (2016). Marriage advantages in perinatal health: Evidence of marriage selection or marriage protection?. Journal of Marriage and Families.

[CR14] Kiernan K, Pickett KE (2006). Marital status disparities in maternal smoking during pregnancy, breastfeeding and maternal depression. Social Science & Medicine.

[CR15] Koski A, Clark S (2021). Child marriage in Canada. Population and Development Review.

[CR16] Luo ZC, Wilkins R, Kramer MS (2004). Disparities in pregnancy outcomes according to marital and cohabitation status. Obstetetrics & Gynecology.

[CR17] Manning WD, Cohen JA (2015). Teenage cohabitation, marriage, and childbearing. Population Research and Policy Review.

[CR18] Marphatia AA, Ambale GS, Reid AM (2017). Women’s marriage age matters for public health: A review of the broader health and social implications in South Asia. Frontiers in Public Health.

[CR19] Raj A, McDougal L, Silverman JG, Rusch ML (2014). Cross-sectional time series analysis of associations between education and girl child marriage in Bangladesh, India, Nepal and Pakistan, 1991-2011. PLoS ONE.

[CR20] Raj A, Saggurti N, Winter M, Labonte A, Decker M, Balaiah D, Silverman JG (2010). The effect of maternal child marriage on morbidity and mortality of children under 5 in India: Cross sectional study of a nationally representative sample. The BMJ.

[CR21] Sawyer SM, Azzopardi PS, Wickremarathne D, Patton GC (2018). The age of adolescence. The Lancet Child & Adolescent Health.

[CR22] Shah PS, Zao J, Ali S (2011). Maternal marital status and birth outcomes: A systematic review and meta-analyses. Maternal and Child Health Journal.

[CR23] Shapiro GD, Bushnik T, Wilkins R, Kramer MS, Kaufman JS, Sheppard AJ, Yang S (2018). Adverse birth outcomes in relation to maternal marital and cohabitation status in Canada. Annals of Epidemiology.

[CR24] Statistics Canada. (2009). *PCCF+ version 5E user*’*s guide*. Ottawa, ON: Statistics Canada.

[CR25] Statistics Canada. (2018). *Postal Code*^*OM*^*Conversion File Plus (PCCF+) version 7B. Reference Guide*. Ottawa, ON: Statistics Canada.

[CR26] UNICEF. (2020). UNICEF data: Monitoring the situation of children and women - Child marriage. https://data.unicef.org/topic/child-protection/child-marriage/#status. Accessed March 8, 2022.

[CR27] United Nations. (2020). Sustainable Development Goals, Goal 5: Achieve gender equality and empower all women and girls. https://www.un.org/sustainabledevelopment/gender-equality/. Accessed March 8, 2022.

